# Hydrogen Bonds with Fluorine in Ligand–Protein Complexes-the PDB Analysis and Energy Calculations

**DOI:** 10.3390/molecules27031005

**Published:** 2022-02-02

**Authors:** Wojciech Pietruś, Rafał Kafel, Andrzej J. Bojarski, Rafał Kurczab

**Affiliations:** Department of Medicinal Chemistry, Maj Institute of Pharmacology, Polish Academy of Sciences, Smetna 12, 31-343 Krakow, Poland; pietrus@if-pan.krakow.pl (W.P.); rafal.kafel@gmail.com (R.K.); bojarski@if-pan.krakow.pl (A.J.B.)

**Keywords:** fluorine, PDB, hydrogen bonds, HBs

## Abstract

Fluorine is a common substituent in medicinal chemistry and is found in up to 50% of the most profitable drugs. In this study, a statistical analysis of the nature, geometry, and frequency of hydrogen bonds (HBs) formed between the aromatic and aliphatic C–F groups of small molecules and biological targets found in the Protein Data Bank (PDB) repository was presented. Interaction energies were calculated for those complexes using three different approaches. The obtained results indicated that the interaction energy of F-containing HBs is determined by the donor–acceptor distance and not by the angles. Moreover, no significant relationship between the energies of HBs with fluorine and the donor type was found, implying that fluorine is a weak HB acceptor for all types of HB donors. However, the statistical analysis of the PDB repository revealed that the most populated geometric parameters of HBs did not match the calculated energetic optima. In a nutshell, HBs containing fluorine are forced to form due to the stronger ligand–receptor neighboring interactions, which make fluorine the “donor’s last resort”.

## 1. Introduction

**Figure 1:**
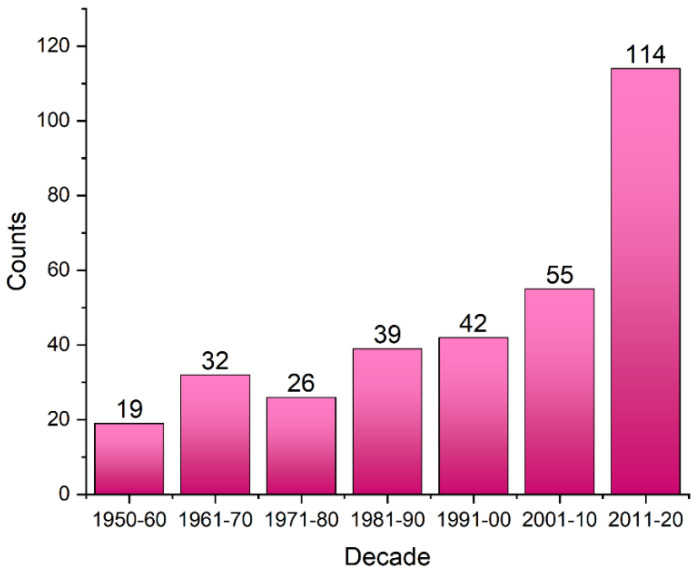
Number of marketed drugs containing fluorine per decade. Data were collected from the DrugCentral 2021 database (accessed 30 April 2021).

Fluorine is the most electronegative element, and this property has a significant impact on the bioavailability, lipophilicity, metabolic stability, acidity/basicity, and toxicity [1]. Since the second half of the 20th century [2], researchers have been exploring the possibility of using fluorinated molecules in medicine [1,3]. The important position of fluorinated molecules in medicinal chemistry can be understood by the exceptionally large number of fluorine-containing drugs currently available on the pharmaceutical market (Figure 1). The share of fluorinated compounds rose from 2% in 1970 to 8% in 1980, 13% in 1990, and reached 18% at the beginning of the 21st century. Among them, six products were in the “top-12” list and employed as anticancer, anti-inflammatory, analgesic, or antidepressant agents in medicine [4]. About 20% of the drugs used in 2010 contained fluorine atom(s) or fluoroalkyl group(s) [3], whereas in the last decade (2011–2020) 114 out of the 410 drugs approved by the Food and Drug Administration (FDA) (data from the Center for Drug Evaluation and Research (CDER)) [5] contained fluorine (Figure 1). Currently, fluorinated pharmaceuticals account for over 50% of the most profitable drugs (blockbuster drugs), and are also recognized as the best among the drugs used in almost all therapeutic areas [6].

The biological activity of drugs is determined by intermolecular interactions. These interactions also play an important role in stabilizing the ligand–biomolecule system. Hydrogen bonds (HBs), in particular, are considered to significantly influence the action of drug molecules on their targets [7,8,9]. Interestingly, fluorine or substituents containing this element have been shown to tune the intermolecular interactions in ligand–protein complexes [1,10]. Although characterized by high electronegativity, fluorine is a weak acceptor of HBs and, unlike other halogens, it is not a halogen bond (XB) donor in aromatic systems [11,12]. However, the results of our previous study on small model systems (e.g., 2,6-difluro-4-halogenoanilines) indicated that fluorine can act as a competitive and attractive acceptor for HBs and XBs as well as form F⋯F interactions [13]. Additionally, it is considered that fluorine-containing HBs are not typical and do not behave like conventional HBs (e.g., O∙∙∙H–O and N∙∙∙H–N), as demonstrated by a more angular nature and preference for less electronegative donors [14].

The biological activity of compounds can be tuned with the use of fluorine. However, there are no rules of thumb for predicting the preferred fluorine substitution sites in a molecule. Despite numerous studies on fluorine, the influence of this element on the pharmacodynamics properties of drugs remains unclear. A statistical analysis of the nature, geometry, and frequency of interactions occurring between fluorine in small molecules and the biological targets included in the Protein Data Bank (PDB) repository may allow understanding of the role of fluorine in ligand–receptor (L–R) complexes. Therefore, we carried out a wide statistical analysis and calculations to quantitatively and qualitatively explore the HBs (contacts) formed with fluorine in biological systems. The findings of this study may contribute to a thorough understanding of the effects of fluorine, to enable its rational use in drug design and for improving the efficiency of computational methods [15,16].

## 2. Results and Discussion 

### 2.1. Choice of a Model System

**Figure 2:**
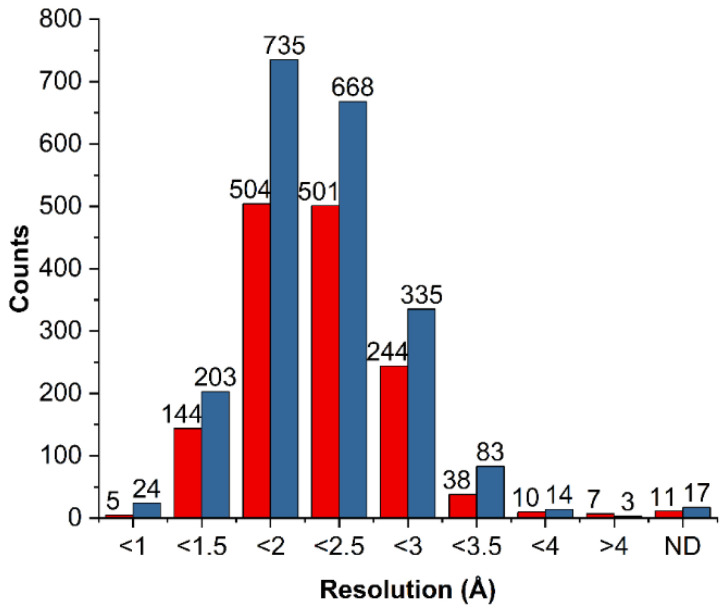
Statistical representation of the spectral resolution of the analyzed crystal structures deposited in the PDB database for L–R complexes with HB containing aliphatic fluorine (red bars) and aromatic fluorine (blue bars). “ND” (not determined) refers to the crystal structures obtained with the methods for which resolution was not specified (e.g., nuclear magnetic resonance).

We carefully chose the model systems used for database mining and quantum chemical calculations, with a focus on providing accurate representations of HBs with fluorine in the biological systems. Three types of HB donors were distinguished, namely hydroxyl, amine, and methyl group, and a pH of 7.4 ± 0.5 was considered to assess the protonation states of all entities. We extracted a ligand with interacting residue for performing high-level quantum chemical calculations in a reasonable time. This allowed us to determine the energy of isolated interaction with fluorine. For extracted amino acids from the main chain, the peptization reaction was reversed. Therefore, to maintain the proper structure of amino acids, missing atoms (hydroxyl to carboxylic group and hydrogen to nitrogen atom) were added and optimized with force field. Since 96% of the analyzed crystal structures were recorded with a resolution of <3 Å (Figure 2), we analyzed all the collected structures.

### 2.2. General Statistics of HBs Containing Fluorine Atoms

It should be emphasized that the thresholds of HB geometric parameters considered in the statistical investigation based on the PDB data can significantly influence the results and conclusion. In this study, we assumed that the HB distance was <4Å and the HB angle was 90°–180°. Because we aimed to determine all contacts with fluorine atoms, these values can significantly exceed the standard geometric parameters of HBs; the distance can be below the sum of the van der Waals radii of interacting atoms, and the angle can differ by up to 120°. Complexes containing ligands with fluorine (from the LigandExpo repository) were extracted and analyzed. If the PDB entry contained more than one asymmetric unit (receptor oligomerization), the number of HBs with fluorine was multiplied by the number of occurrences of the same ligand. All measured HBs were used in further analysis, even if they came from the same PDB entry. The ligands were divided into two categories: molecules in which fluorine is bonded to an aliphatic carbon (F_al_) and molecules containing fluorine bonded to an aromatic carbon (F_ar_).

**Figure 3:**
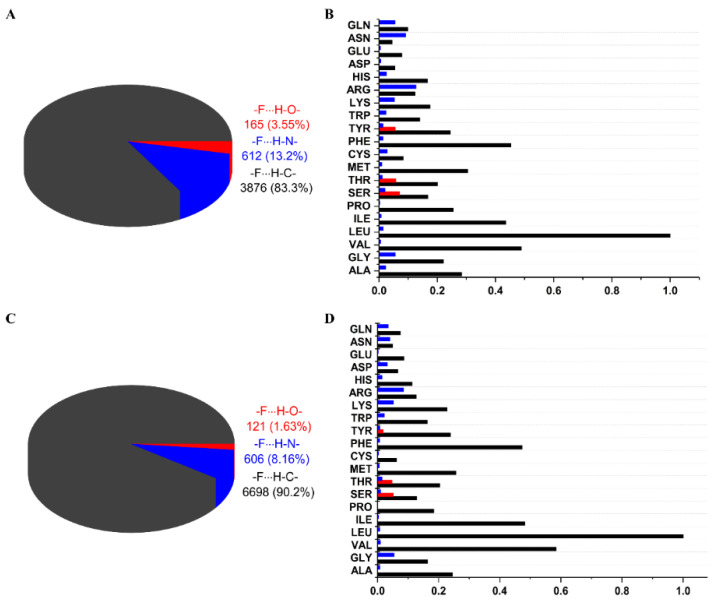
Number of hydrogen bonds found in the PDB repository to meet the boundaries for geometric parameters: (**A**) between F_al_ and OH, NH, and CH donors; (**B**) normalized division into interacting amino acids; (**C**) between F_ar_ and OH, NH, and CH donors; and (**D**) normalized division into interacting amino acids.

A total of 1787 (F_al_) and 2324 (F_ar_) unique PDB entries were found for fluorine-containing ligands. Based on the assigned boundaries and defined geometric thresholds, 165 interactions with a hydroxyl group, 612 with an amine group, and 3875 with a methyl group were identified for aliphatic fluorine (Figure 3A); and 121 interactions with a hydroxyl group, 606 with an amine group, and 6698 with H–C were identified for aromatic fluorine (Figure 3C). The number of F⋯H–O and F⋯H–N HBs was found to be larger for F_al_, whereas the number of F⋯H–C HBs was two-fold higher for F_ar_. For OH donors, three amino acids (SER, THR, TYR) were identified to be involved in HBs with F_al_; however, for F_ar_, it appeared that TYR participates less frequently in HBs, which may be attributed to the greater acidity of the OH group.

The more significant differences were observed for NH donors, in which the amino acids commonly involved in HBs with F_al_ were in the order ARG>ASN>GLN>GLY>LYS (Figure 3B) and in HBs with F_ar_ were in the order ARG>GLY>LYS>GLN>ASN (Figure 3D). Surprisingly, glycine is the second most common amino acid, forming HBs with F_ar_ because the others are polar amino acids with a free amino group in their side chain.

By contrast, no clear preferences of amino acids in the occurrence of HBs with F_al_ and F_ar_ were observed in the case of CH donors. However, the results highlighted that fluorine most frequently forms HBs with nonpolar amino acids (LEU, VAL, PHE, ILE, ALA), implying that it prefers hydrophobic areas of binding pockets (Figure 3B,D).

**Figure 4:**
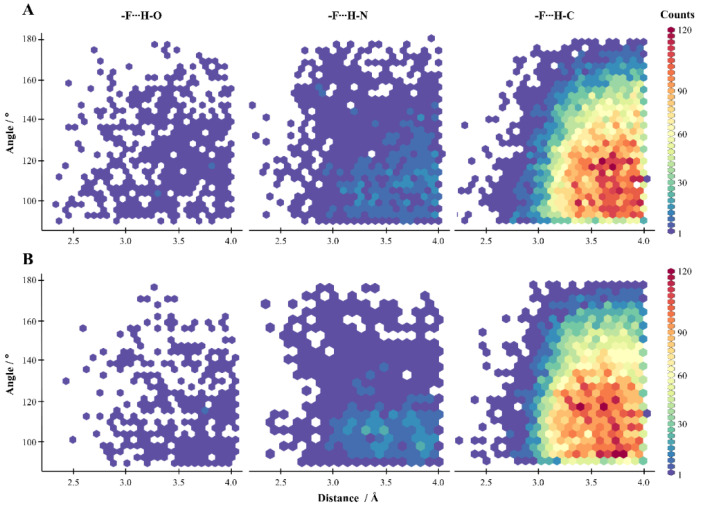
Density maps of geometric parameters of HBs with (**A**) F_al_ and (**B**) F_ar_.

Based on this classification, we generated the density maps showing the geometric parameters of HBs (Figure 4). As only a small number of F⋯H–O HBs were identified, certain conclusions could not be drawn (Figure 4). The density maps of HB geometric parameters obtained for the NH and CH donors (as well as OH) revealed that fluorine prefers geometries with a distance of >3 Å and an angle of 100°–140° (Figure 4). However, it should be noted that more HBs were found for NH than OH donors, with a more linear geometry and short distances, but in many cases, those interactions are forced by the neighboring functional groups of a ligand interacting with amino acids.

In summary, fluorine-containing HBs reveal more angular geometric preferences than typical HBs (rather linear HBs O⋯H–O, N⋯H–N). Thus, in the next step, we explored the relationship between the geometry of F⋯H–X (X = O, N, C) bond and the energy contribution to the ligand–receptor complexes.

### 2.3. Energy of HBs with Fluorine

Ligand–receptor complexes are stabilized by various intermolecular forces, such as strong HBs (O∙∙∙H–O, N∙∙∙H–O, N∙∙∙H–N), weak HBs (O∙∙∙H–C, S∙∙∙H–N), XBs, π-stacking, salt bridge, amide stacking, cation-π, and hydrophobic interactions and others [17]. Fluorine-containing HBs, especially those with hydroxyl and amine donors, are not common in biological systems (Figure 3 and Figure 4). Therefore, it is important to determine the strength and geometric preferences of these HBs in biological systems. In this study, we attempted to evaluate the nature and energetic dependencies of HBs with fluorine in the theoretical background by performing quantum chemical calculations using small molecular systems extracted from ligand–biomolecule crystals (O–H, N–H, +N–H, and C–H were only considered to be HB donors). We determined the energy of HBs with fluorine found in crystal structures by applying three different methods as follows: (1) Diff—energy was calculated as the difference between the energy of the interacting molecules and the sum of the energies of isolated species calculated in Gaussian; (2) QTAIM—energy was calculated at BCP in AIMAll software; and (3) ETS—energy was calculated between two interacting molecules using the ETS-NOCV scheme implemented in ADF software.

At first, a simple statistical analysis was performed on the data obtained from the three approaches using the correlation coefficient and Pearson test in R (Supplementary Tables S1 and S2). The results of the analysis indicated the highest correlation between Diff and ETS methods (*p* < 0.05, correlation coefficient ~1) because they consider the energy of the entire system and approximately 70% of the calculated energy accounts for the same nature of interaction (attractive/repulsive). Additionally, the correlation decreased for stronger interactions (Supplementary Tables S1 and S2) due to the fact that the Diff method is intended for weak and medium HBs; strong HBs result in geometry distortion of the interacting molecules, which decreases the accuracy of the evaluation of the HB itself [18]. The energies of HBs calculated by QTAIM did not correlate with those determined by the remaining methods, since this method takes only isolated L–R interaction and neglects long-range interactions occurring between the atoms from separated fragments.

**Figure 5:**
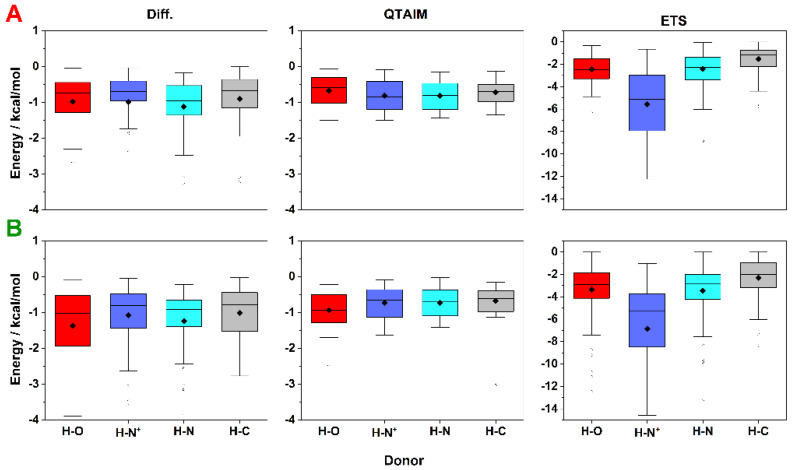
Box plots showing the distribution of the stabilization energy for HBs containing fluorine bonded to an (**A**) aliphatic or (**B**) aromatic carbon. A comparison is made for the individual donor groups (OH, ^+^NH, NH, and CH) as well as the calculation approaches used (Diff, QTAIM, and ETS).

The distribution of calculated interaction energies for all selected complexes for a given type of HB with fluorine and method is illustrated in Figure 5. For HBs with F_al_, the interaction energy calculated by QTAIM varied between 0 and −1.2 kcal/mol (the weakest HBs were found for F_al_⋯H–O, with an energy value of −0.64 kcal/mol). For F_al_⋯H–C HBs, the energy was (−0.69 kcal/mol). The strongest HBs (~−0.8 kcal/mol) were observed for F_al_⋯H–N (no significant difference was noted between F_al_⋯H–N HB and F_ar_⋯H–N^+^ HB) (Figure 5A). The energy range determined by the Diff method was also between 0 and –1.2 kcal/mol, while the energy determined by the ETS method ranged from 0 to −8 kcal/mol (Figure 5A). Additionally, the Diff method indicated that the charge-assisted F_al_⋯H–N^+^ was the weakest HB (−0.70 kcal/mol), while F_al_⋯H–N HB was stronger (−0.96 kcal/mol). Unlike Diff, the results of the ETS method showed that the charge-assisted F_al_⋯H–N^+^ HB was the stronger (−5.11 kcal/mol), while F_al_⋯H–N HBs were weaker than F_al_⋯H–O HBs (−2.28 and −2.46 kcal/mol, respectively) (Figure 5A). For HBs with F_ar_, a different trend was noted in the QTAIM method than for HBs with F_al_, where the strongest HBs had OH as a donor (−0.94 kcal/mol). The F_ar_⋯H–N HBs were found to be slightly weaker with median energy values of ~–0.7 kcal/mol (for ^+^NH and NH donors), and the weakest was F_ar_⋯H–C HB (−0.62 kcal/mol) (Figure 5B). A comparison of Diff and ETS methods revealed a similar trend as in the case of F_al_—the Diff method indicated that the F_ar_⋯H–N^+^ HB was the weakest (−0.8 kcal/mol), while the ETS method indicated it as the strongest interaction (−5.3 kcal/mol) (Figure 5B). The QTAIM method showed that F⋯H–O HBs with F_ar_ were stronger than those with F_al_, while HBs with other donors were found at a similar energy level. However, it should be emphasized that both Diff and ETS methods revealed higher stabilization energy for HBs with F_ar_ compared to HBs with F_al_.

**Figure 6:**
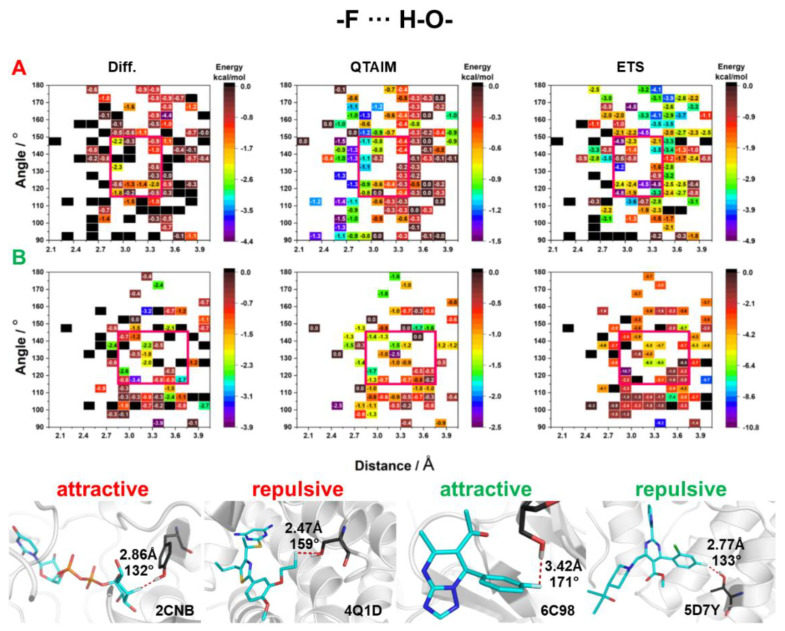
HB energy maps generated based on Diff, QTAIM, and ETS calculations of interaction energy between OH donor and fluorine attached to (**A**) an aliphatic fragment and (**B**) an aromatic ring for specific geometric parameters. The areas for which the highest stabilizing energy was observed in all three methods are marked with a red square.

The hydroxyl donor occurs in the side chains of three amino acid—tyrosine (TYR), threonine (THR), and serine (SER). The phenolic hydroxyl group (TYR) is significantly more acidic (pK_a_ of about 9.8 in polypeptides) than the aliphatic hydroxyl group (SER or THR, pK_a_ ~13.6) [19]. In addition, Graton et al. found in an analysis of the PDB repository that the distances and angle of HBs with a hydroxyl group decreased in the order THR > SER > TYR, which suggests that TYR forms the stronger HBs [20]. In the present study, the results obtained by the QTAIM method revealed that for F_al_ (Figure 6A), the energy of HBs does not depend on the F⋯H–O angle, as the highest values were observed in the whole range of the analyzed angles. Instead, the energy of F_al_⋯H–O HBs closely correlated with the distance, as observed in the case of conventional hydrogen bonding. It should be mentioned that the QTAIM method showed higher energy for F⋯H–O HBs with aromatic fluorine than for HBs with aliphatic fluorine (Figure 6). Most of the F⋯H–O HBs with distances shorter than 2.75 Å are repulsive (Figure 6), which shows that despite the stabilizing nature of the F⋯H–O HB itself, the interacting fragments have a positive energy contribution (repulsive character). This effect may be due to the interaction of the neighboring atoms, or high positive kinetic energy. All three methods showed that the highest stabilizing energies (red squares in Figure 6) were in the range of 2.85–3.45 Å (for both fluorine) and 150°–120° for F_al_ and 145°–120° for F_ar_, suggesting that these are the optimal ranges of geometric parameters for F⋯H–O HBs. The analysis of the selected crystal structures did not show any significant differences between the F⋯H–O HBs of attractive and repulsive nature. The only differentiating factor identified was the F∙∙∙O distance (Figure 6).

**Figure 7:**
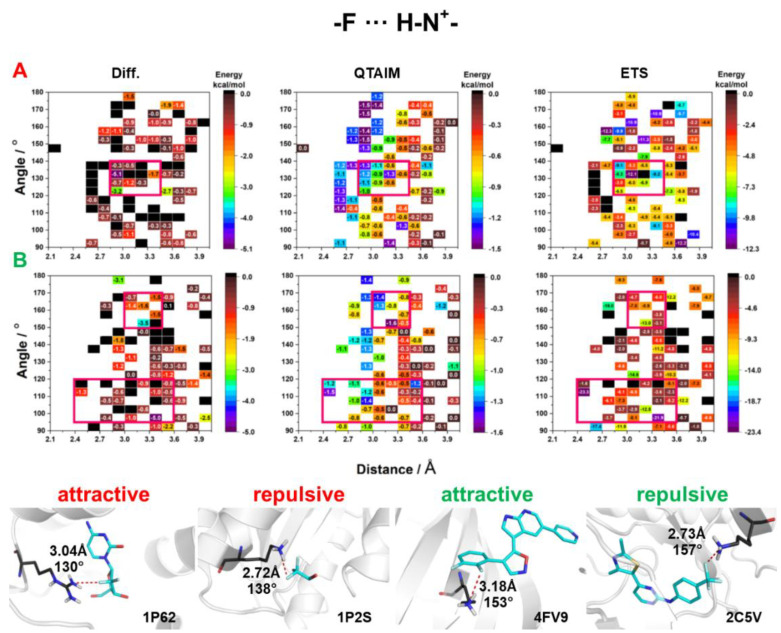
HB energy maps generated based on Diff, QTAIM, and ETS calculations of interaction energy between positively charged NH donor and fluorine attached to (**A**) an aliphatic fragment and (**B**) an aromatic ring for specific geometric parameters. The areas for which the highest stabilizing energy was observed in all three methods are marked with a red square.

Among amino acids, three (ARG, LYS, HIS) have additional amino groups. The side chain of arginine (ARG) is amphipathic because at physiological pH it contains a positively charged guanidine group (pK_a_ = 12.48). Another amphipathic amino acid is lysine (LYS), the side chain of which contains a positively charged primary amine group at the end of the long hydrophobic carbon tail (pK_a_ = 10.53). Histidine (HIS) contains an imidazole side chain. His pK_a_ is 6, above which one of the two protons is missing (in physiological pH, histidine has two tautomers). Since it is difficult to automatically protonate the appropriate nitrogen atom of histidine which forms an F⋯H–N^+^ HB, we calculated the energy of F⋯H–N^+^ HB only for LYS and ARG (Figure 7). The QTAIM calculations showed that the energy of F_al_⋯H–N^+^ and F_al_⋯H–O HBs was similar. Additionally, F⋯H–N^+^ was found to be strongly influenced by the distance between F∙∙∙N and not by the angle (Figure 7) (as noticed for F⋯H–O HBs). A similar trend for both aliphatic and aromatic F was observed for F⋯H–N^+^ HBs, but the highest interaction energy was mostly localized at higher values of the F_ar_∙∙∙H–N^+^ angle (Figure 7). For aliphatic fluorine, the range of geometric parameters in which the three methods indicated the strongest F_al_∙∙∙H–N^+^ HBs was 140°–120° and 2.85 –3.45 Å (as for F⋯H–O HB). In the case of F_ar_, two ranges of geometric parameters were distinguished: (1) 170°–150° and 3.0–3.6 Å; and (2) < 120° and 2.4–3.6 Å. Due to its large volume, the guanidine group interacts not only with fluorine directly but also with neighboring atoms. Therefore, the energy calculated by Diff and ETS methods might be overestimated.

Interestingly, the analysis of crystal structures with F⋯H–N^+^ HBs showed that for F⋯N^+^ distances of <2.8 Å, almost 70% of HBs exhibited a destabilizing character. Moreover, the interaction of positively charged nitrogen with the CF_3_ group, with a partial positive charge on the carbon atom, is often repulsive (Figure 7).

**Figure 8:**
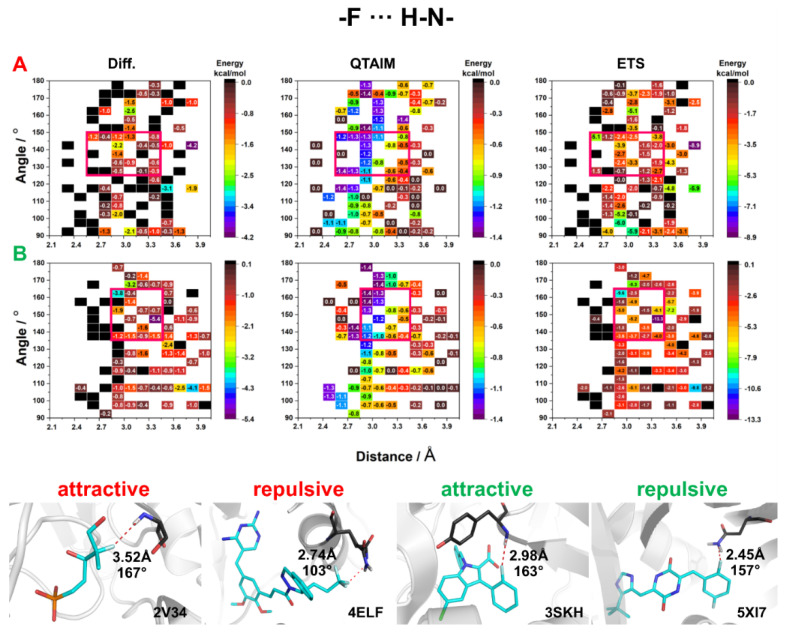
HB energy maps generated based on Diff, QTAIM, and ETS calculations of interaction energy between NH donor and fluorine attached to (**A**) an aliphatic fragment and (**B**) an aromatic ring for specific geometric parameters. The areas for which the highest stabilizing energy was observed in all three methods are marked with a red square.

The F⋯H–N HBs were found almost four times more frequently in PDB than F⋯H–O HBs (Figure 3 and Figure 4). The energies of F⋯H–N HBs were calculated for all amino acids, except for arginine and lysine as these amino acids contain positively charged nitrogen atoms. Furthermore, whether the nitrogen atom was in the main chain or the side chains (ASN, GLN) was not considered in the analysis. The energies of F⋯H–N HBs determined by the QTAIM method showed no significant differences between F_al_ and F_ar_. In addition, it must be noted that energy is inversely proportional to the HB distance and does not depend on the F⋯H–N angle (Figure 8). However, since the nitrogen atom was mostly present in the main chain, and was thus adjacent to different atoms, the energies calculated by Diff and ETS methods mostly had a destabilizing nature, which might be due to steric effects. On the other hand, for F_al_, the areas where the energies were found to be high and exhibited a stabilizing character had a narrow range of 150°–125° and 2.4–3.45 Å, while for F_ar_ the areas were within the range of geometric parameters (165°–135° and 2.85–3.75 Å) (Figure 8). The analysis of selected crystal structures showed no significant differences between the systems with attractive energy and repulsive energy (Figure 8).

**Figure 9:**
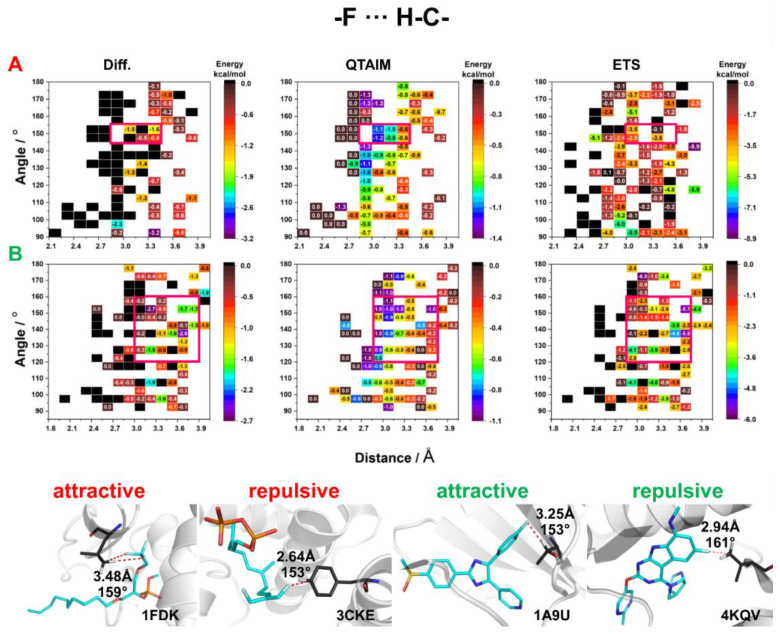
HB energy maps generated based on Diff, QTAIM, and ETS calculations of interaction energy between CH donor and fluorine attached to (**A**) an aliphatic fragment and (**B**) an aromatic ring for specific geometric parameters. The areas for which the highest stabilizing energy was observed in all three methods are marked with a red square.

The F⋯H–C HBs were found to be the most abundant in biological systems (Figure 3 and Figure 4). The interaction energies calculated by the QTAIM method showed that F_al_⋯H–C HBs are stronger than F_ar_⋯H–C HBs. In addition, the interactions with an HB distance of <2.7 Å showed a destabilizing character (Figure 9). The results produced by Diff and ETS methods were quite divergent, and it is difficult to find any constant trend. Interestingly, the results obtained from all three methods indicate that HBs with F_al_ are stronger than those with F_ar_ (Figure 9). The energetically favorable HBs with F_al_ had an angle of 155°–145° and a distance of >2.7 Å, while HBs with F_ar_ had an angle of 160°–120° and a distance of >2.85 Å (red squares in Figure 9). The analysis of selected crystal structures showed that F⋯H–C HBs mostly exhibited a stabilizing character for distances longer than 3 Å (Figure 9).

Analysis of the ETS-NOCV decomposition results showed that for uncharged donors (OH, NH, CH) the contribution of Coulomb energy term has the greatest impact on the stabilization energy of HBs with fluorine, while for NH^+^ the XC energy term has the largest contribution. The reason for the destabilizing nature of the shorter HBs with fluorine may be due to the high value of the kinetic energy contribution (Figure S2). To determine the significance of HBs with fluorine, the density maps of geometric parameters of HBs found in the PDB repository (Figure 3 and Figure 4) were compared with the corresponding HB energy maps (Figure 6, Figure 7, Figure 8 and Figure 9). The proposed areas (red squares) of favorable geometric parameters with the highest energy values did not match with the most occupied areas of geometric parameters. This suggests that HBs with fluorine do not play a significant role in the stabilization of the L–R system and are often formed under unfavorable geometric parameters.

## 3. Materials and Methods

### 3.1. PDB Analysis

We performed a statistical analysis of the structural data and investigated in detail the geometric parameters of the intermolecular HBs of fluorine in the structures deposited in the PDB repository. In the first step, all fluorine-containing ligands were identified in the LigandExpo database [21] and then divided into two groups: fluorine attached to an aliphatic carbon (F_al_) and fluorine attached to an aromatic carbon (F_ar_). In the next step, all crystal structures containing the abovementioned ligands were identified. The positions of hydrogen atoms were added, considering the stereochemical rules determining the most favorable position of hydrogens, using the Protein Preparation Wizard (Schrödinger Maestro Software) [22]. The in-house script was used to detect the interactions (contact) occurring between fluorine (as an acceptor) in the ligand and the neighboring HB donors (i.e., OH, NH, CH) in the receptor that met the following criteria: a distance of <4 Å and an angle of 90–180°. 

Based on the obtained data, density maps showing the distribution of the geometric parameters of HBs were generated using R [23] environment as well as RColorBrewer [24], Hexbin [25], Rbokeh [26], and ggplot2 [27] libraries.

### 3.2. Calculation of Interaction Energy

To determine the energy of the studied intermolecular interactions with fluorine, all complexes obtained from the PDB were divided into subgroups based on the following: (i) angle of HB (ranged from 90° to 180° with a step of 10°), (ii) distance of HB (ranged from 2.5 to 4 Å with a step 0.1 Å), (iii) donor type of HB (OH, NH, ^+^NH, CH), and (iv) whether fluorine is bonded to an aromatic (F_ar_) or aliphatic (F_al_) carbon. Then, one representative PDB complex was randomly selected from each rectangle defined by unit distance and angle change. In the next step, all the selected systems were visually inspected to identify those in which the HB with fluorine was not the main stabilizing interaction and the number of adjacent supporting interactions was the smallest. These identified complexes were used to calculate the interaction energy of the fluorine-containing HB for the given geometric parameters. Figure S1 illustrates the distribution of complexes in a given HB distance–angle interval.

The appropriate ionization states at pH 7.4 ± 0.5 were determined using Epik v3.4 [28,29]. Using an in-house script, the structure of the ligand and the amino acid participating in the interaction was extracted, the missing atoms (included in the peptide bond) were added, and their positions were optimized (OPLS3 force field). The interaction energy of the identified complexes with fluorine was calculated using three commonly used approaches as follows.

The first method, named difference approach or Diff, works based on the assumption that the total interaction energy equals the energy required to separate two interacting molecules. Thus, the energy between the HB donor (X) and the acceptor (Y) is calculated as the difference between the total energy of the X∙∙∙Y complex and the sum of the total energies of its frozen components [18,30]:

$$E_{int}=E\left(X\cdot Y\right)-\left[E\left(X\right)+E\left(Y\right)\right]$$

The energies of the separated molecules, as well as that of the complex, were calculated in Gaussian G16 software [31], using the Minnesota functional M06-2X [32,33] and Karlsruhe basis set def2-TZVP [34]. The polarizable continuum model (PCM) (solvent = water) was used for the calculation [35,36].

The second approach works based on Bader’s quantum theory of “atoms in molecules” (QTAIM). In this approach, the topological analysis of electron density was carried out in AIMAll program [37]. The electron density calculated in Gaussian G16 at the M06-2X/def2-TZVP level and the PCM (solvent = water) were used in the analysis. The energy of the noncovalent bonds detected in the crystal structures was calculated using the Espinosa equation as follows:

$$E_{int}=\frac{1}{2}v\left(r\right)$$

where *E_int_* is the energy of the interatomic interaction and *v*(*r*) is the kinetic energy at the bond critical point (BCP). The above equation can be used for all types of HBs, van der Waals interactions, and weak interactions such as H∙∙∙H and C–H∙∙∙O [38].

The third approach works based on the energy decomposition analysis. Bonding was analyzed using the extended transition state (ETS) method [39], with the natural orbitals for the chemical valence (NOCV) scheme [40,41,42]. In this approach, the total energy of bonding between the interacting molecules (ΔE_int_) is divided into different components as follows:

(1) $$\Delta E_{int}=\Delta E_{dist}+\Delta E_{el}+\Delta E_{Pauli}+\Delta E_{orb}$$

where Δ*E_dist_* is the energy required to promote the separated fragments from their equilibrium geometry to the structure they will take up in the complex, Δ*E_e_*_l_ is the energy of the electrostatic interaction occurring between the two fragments in the supermolecule geometry, Δ*E_Paul_*_i_ is the energy of repulsion between the occupied orbitals of the two fragments, and Δ*E_orb_* or the orbital interaction term refers to the energy of the stabilizing component due to the final orbital relaxation. All calculations were performed using the Amsterdam Density Functional (ADF) program [43,44,45,46], using the ETS-NOCV scheme. The Becke, Lee, Yang, and Parr exchange-correlation functional with the Grimme dispersion correction (B3LYP-D3) was used. A standard double-ζ STO basis containing one set of polarization functions was adopted for all the electrons (TZP). The total (electronic) bonding enthalpies (ΔE = Δ*E_int_*) did not include the zero-point energy (ZPE) additions, finite temperature contributions or basis set superposition error corrections (BSSE).

## 4. Conclusions

Fluorine is a common substituent in medicinal chemistry and is found in the structure of several currently available blockbuster drugs. This element influences many pharmacokinetic and pharmacodynamic properties of drugs, but its role in stabilizing ligand–biomolecule systems still remains unclear. In this study, we performed a statistical and theoretical analysis of HBs with fluorine found in the PDB database, focusing on the different HB donors (hydroxyl, amine, and methyl groups). The energy range of distinct HBs (i.e., F⋯H–O, F⋯H–N^+^, F⋯H–N, F⋯H–C) and optimal ranges of geometric parameters of HBs with fluorine were determined based on the selected PDB complexes.

The results of the analyses showed significant differences in the interaction of fluorine attached to an aliphatic carbon (F_al_) and fluorine attached to an aromatic carbon (F_ar_). The F⋯H–O HBs with F_ar_ are more frequently formed with SER and THR, while those with F_al_ are formed by all amino acids with a polar hydroxyl group. Typically, F⋯H–N HBs are formed with amino acids that have an amino group in their side chain (ARG, LYS, ASN, GLU). Hydrophobic amino acids most often form F⋯H–C interactions, which suggests that fluorine prefers a hydrophobic environment in biological systems. It is worth noting that due to the three free electron pairs of fluorine, HBs are only influenced by the donor–acceptor distance and not by the angles. Although the three free electron pairs occupy the entire space around fluorine, F⋯H–X HBs exhibit the characteristics of HBs, with exceeded standard angles. However, no significant differences were noted in the energies of HBs with fluorine depending on the donor type, which indicates that fluorine acts as a weak HB acceptor for all types of atoms. The optimal ranges of geometric parameters for HBs with fluorine were found to be 150°–120° and 2.9–3.6 Å. For F⋯N^+^ interactions, an HB distance shorter than 2.8 Å showed a destabilizing character in almost 70% of the cases.

It must be emphasized that all the analyzed crystal structures may not be crystallized at the lowest free energy form, and hence the observed interactions might not be in optimal geometries [47]. However, the results suggest that HBs with fluorine are forced to form, due to the stronger ligand–receptor neighboring interactions, which make fluorine the “donor’s last resort” [48]. This is in line with Margareth Etter’s rule that stronger HBs form first, and weaker donors and acceptors interact afterward [47]. All these findings suggest that fluorine does not form strong, stabilizing intermolecular interactions, and thus it seems that indirect influence of this element (electrostatic, inductive, and resonance effects) has a greater impact on the biological activity of compounds than his influences on the pharmacodynamics. The results of this study may contribute to a thorough understanding of hydrogen bonding with fluorine in biological systems which may serve to improve the tools currently available for the rational design of new fluorinated drugs.

## Data Availability

The data were obtained from the PDB repository and the DrugCentral 2021 database (accessed 30 April 2021).

## Supplementary Materials

The following supporting information can be downloaded, Figure S1: Points of geometrical parameters of hydrogen bonds for which the energy value was calculated; Figure S2: Box plots showing the distribution of the different energy components: kinetic (red), electrostatic (cyan), Coulomb (blue), XC (olive), dispersion (magenta) energy for HB donors and fluorine attached to (A) an aliphatic fragment and (B) an aromatic ring. The energy values were calculated for specific geometric parameters using ETS-NOCV approach. Table S1: Correlation coefficient values calculated between results from every method; Table S2: Pearson test values calculated between results from every method.

## Sample Availability

Samples of the compounds are not available from the authors.
