# Supplementary material for: Hydrogen Bonds with Fluorine in Ligand–Protein Complexes-the PDB Analysis and Energy Calculations
Source: Molecules. 2022 Feb 2;27(3):1005. doi: 10.3390/molecules27031005 (PMC8838457; doi:10.3390/molecules27031005)
Supplement: Supplementary file 1 [file molecules-27-01005-s001.zip › molecules-1555706-supplementary.pdf]

Supplementary information

Hydrogen bonds with fluorine in ligand-protein complexes – the PDB analysis and energy calculations

Wojciech Pietruś [1], Rafał Kafel [1], Andrzej J. Bojarski [1], Rafał Kurczab [1,*]

[1] Department of Medicinal Chemistry, Maj Institute of Pharmacology, Polish Academy of Sciences, Smetna 12, 31-343 Krakow, Poland1; pietrus@if-pan.krakow.pl (W.P.); bojarski@if-pan.krakow.pl (A.J.B.)

* Correspondence: kurczab@if-pan.krakow.pl; Tel.: +48-126-62-3301

Table S1. Correlation coefficient values calculated between results from every method.

	$F_{ar}\cdots H\text{-}C$				$F_{al}\cdots H\text{-}C$		
Methods	Diff	QTAIM	ETS		Diff	QTAIM	ETS
**Diff**	1.00	0.07	0.72	Diff	1.00	0.04	0.52
**QTAIM**	0.07	1.00	0.27	QTAIM	0.04	1.00	0.21
**ETS**	0.72	0.27	1.00	ETS	0.52	0.21	1.00
	$F_{ar}\cdots H\text{-}N$				$F_{al}\cdots H\text{-}N$		
	Diff	QTAIM	ETS		Diff	QTAIM	ETS
**Diff**	1.00	0.05	0.76	Diff	1.00	0.15	0.83
**QTAIM**	0.05	1.00	0.11	QTAIM	0.15	1.00	0.09
**ETS**	0.76	0.11	1.00	ETS	0.83	0.09	1.00
	$F_{ar}\cdots H\text{-}N^+$				$F_{al}\cdots H\text{-}N^+$		
	Diff	QTAIM	ETS		Diff	QTAIM	ETS
**Diff**	1.00	0.16	0.40	Diff	1.00	0.04	0.33
**QTAIM**	0.16	1.00	0.22	QTAIM	0.04	1.00	0.02
**ETS**	0.40	0.22	0.00	ETS	0.33	0.02	1.00
	$F_{ar}\cdots H\text{-}O$				$F_{al}\cdots H\text{-}O$		
	Diff	QTAIM	ETS		Diff	QTAIM	ETS
**Diff**	1.00	0.21	0.41	Diff	1.00	0.02	0.69
**QTAIM**	0.21	1.00	0.30	QTAIM	0.02	1.00	-0.04
**ETS**	0.41	0.30	1.00	ETS	0.69	-0.04	1.00

Table S2. Pearson test values calculated between results from every method.

**Methods**	**Methods**	***p***	**Methods**	**Methods**	***p***
$F_{ar}\cdots H\text{-}C$			$F_{al}\cdots H\text{-}C$		
ETS	Diff	4.22E-05	ETS	Diff	2.82E-08
QTAIM	ETS	2.90E-02	QTAIM	ETS	2.58E-02
Diff	QTAIM	1.26E-01	Diff	QTAIM	7.10E-01
$F_{ar}\cdots H\text{-}N$			$F_{al}\cdots H\text{-}N$		
ETS	Diff	4.36E-05	ETS	Diff	2.20E-16
QTAIM	ETS	4.02E-03	QTAIM	ETS	3.22E-01
Diff	QTAIM	4.12E-02	Diff	QTAIM	9.50E-02

$F_{ar}\cdots H\text{-}N^+$			$F_{al}\cdots H\text{-}N^+$		
ETS	Diff	2.20E-16	ETS	Diff	7.52E-04
QTAIM	ETS	2.19E-03	QTAIM	ETS	8.55E-01
Diff	QTAIM	4.57E-01	Diff.	QTAIM	7.20E-01
**$F_{ar}\cdots H\text{-}O$**			**$F_{al}\cdots H\text{-}O$**		
ETS	Diff	2.20E-16	ETS	Diff	2.20E-16
QTAIM	ETS	2.62E-01	QTAIM	ETS	4.09E-01
Diff	QTAIM	6.47E-01	Diff	QTAIM	8.00E-01

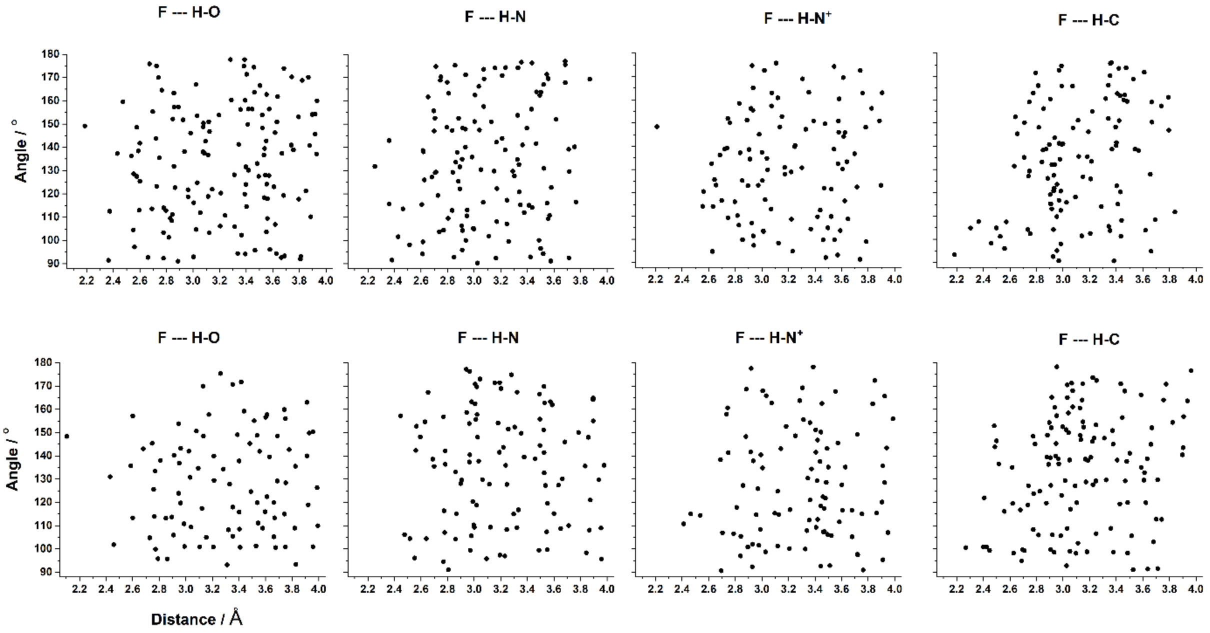

Figure S1. Points of geometrical parameters of hydrogen bonds for which the energy value was calculated.

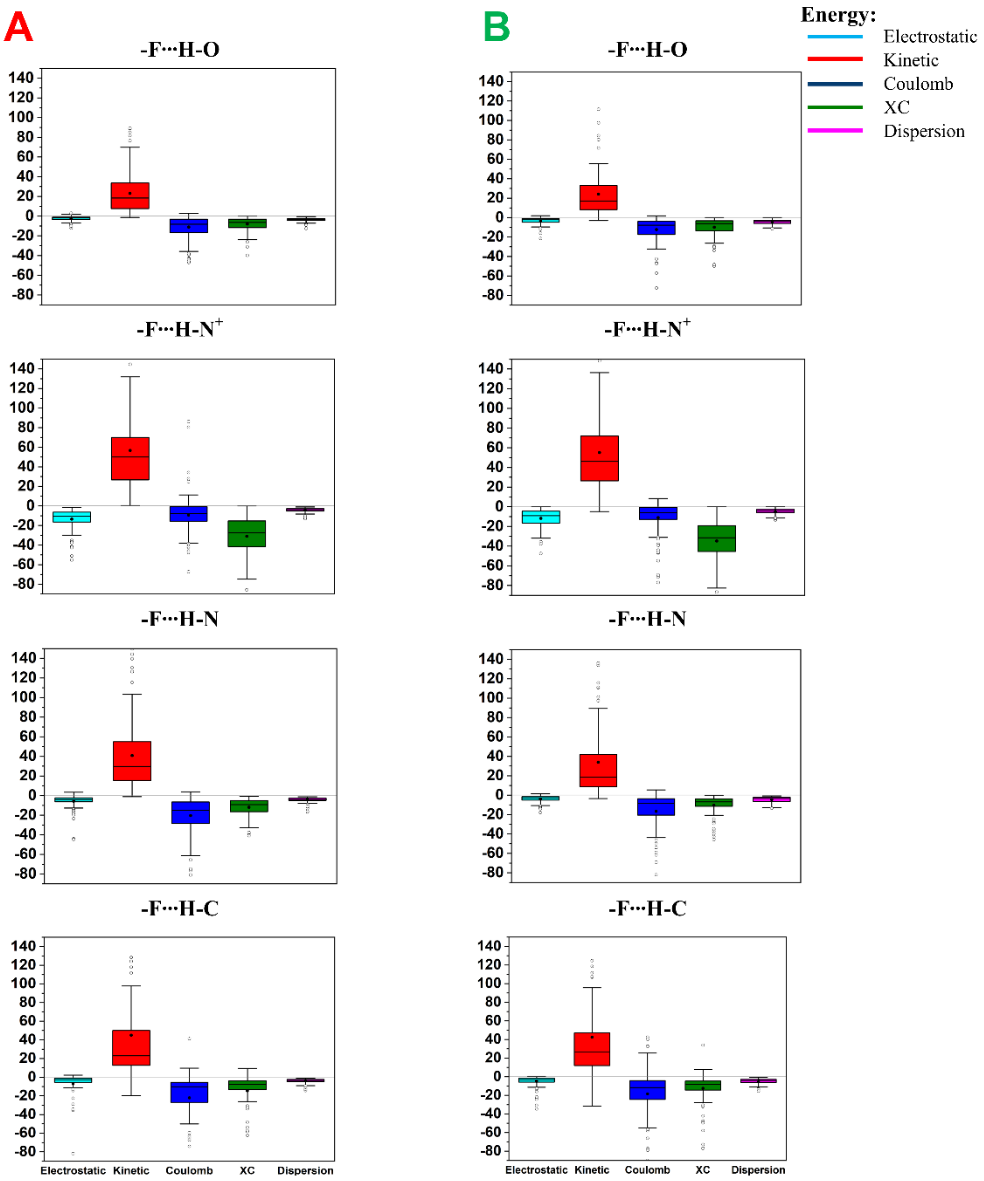

Figure S2. Box plots showing the distribution of the different energy components: kinetic (red), electrostatic (cyan), Coulomb (blue), XC (olive), dispersion (magenta) energy for HB donors and fluorine attached to (A) an aliphatic fragment and (B) an aromatic ring. The energy values were calculated for specific geometric parameters using ETS-NOCV approach.
